# Reflecting through menopause: introducing the MIRROR cycle to support reflection during the menopause transition

**DOI:** 10.1177/17449871261446828

**Published:** 2026-06-05

**Authors:** Camille Cronin, Sara Donevant, Kerri-ann Hughes, Marja Kaunonen, Jette Marcussen, Rhonda Wilson

**Affiliations:** Independent Consultant/Professor of Nursing, Adjunct Professor, RMIT, Australia; Visiting Fellow, University of Essex, UK; Associate Professor, University of South Carolina, College of Nursing, USA; Associate Professor, School of Nursing, College of Health, Massey University, New Zealand; Emerita Professor, Faculty of Social Sciences, Tampere University, Tampere, Finland; Associate Professor, Research unit OPEN, Department of Clinical Research, University of Southern Denmark; Head of Nursing Research, Department of Dermatology, Zealand University Hospital; Docent, Centre for Health, University College Absalon, Denmark; Professor of Nursing, Department of Nursing, RMIT University, Australia

**Keywords:** menopause, nurses, reflection, reflective practice, women’s health, professional development, workforce and employment

## Abstract

**Background:**

The menopause transition is a complex biopsychosocial life-course event that often coincides with significant role changes, including caregiving responsibilities, evolving family dynamics and sustained workplace participation. Many women describe menopause as disruptive, isolating and threatening to identity, with implications for well-being, work performance and professional engagement.

**Aim:**

This paper argues for the integration of reflection into nursing practice, including education, clinical encounters and workplace well-being initiatives, to better support women in healthcare who are approaching or experiencing the menopause transition.

**Methods:**

Peer-reviewed and key grey literature examining menopause-related workplace impacts, healthcare gaps and service provision were reviewed. Established reflective practice models were synthesised and adapted, informed by the authors’ body of work on menopause and the nursing workforce.

**Results:**

An integrated menopause-specific reflective framework, the MIRROR Cycle, was developed. The framework outlines practical reflective strategies including journaling, reflective supervision, Schwartz rounds, structured debriefing, narrative work, mindfulness and appraisal checklists. Socio-cultural and systemic barriers including stigma, silence, fear, limited training, fragmented services and organisational ageism and sexism were identified as factors compounding menopausal distress.

**Conclusion:**

Embedding the MIRROR Cycle across education, clinical care and workplace policy may enhance coping, identity integration and organisational responsiveness, supporting inclusive and menopause-informed nursing practice.

## Introduction

The perimenopausal transition reflects a progressive loss of ovarian follicular function, which culminates in menopause, clinically defined as the permanent cessation of menstruation. These changes bring an array of symptoms that impact the body including vasomotor, psychosocial, cognitive and urogenital being the most common (Kalita et al., 2024; Peters et al., 2025). For many women, these symptoms are episodic and manageable, but for a substantial growing minority they are uncomfortable, sometimes persistent and debilitating, with real consequences for everyday well-being, relationships and employment (Martin-Key et al., 2025). In parallel, societal silence and workplace cultures that minimise mid-life health contribute to isolation and shame (Riach and Jack 2021). Reflective practice offers a structured route for women, clinicians and organisations to make sense of experiences, identify needs and co-design health and well-being support for sustained participation at work and home life.

This paper focuses on women, with particular attention to nurses navigating the menopause transition. It sets out why reflective practice matters during menopause; introduces a practical, adaptable reflection framework tailored to menopausal experience and offers strategies to pause, take stock and reflect at individual, team and organisational levels. The paper also examines the social, clinical and structural factors that make menopause particularly challenging and concludes with recommendations for nursing practice, workplace policy and service design. The argument is grounded in reflective practice literature alongside recent empirical and grey literature documenting lived experience, workplace impact and service gaps related to menopause.

## What is reflection?

Reflection is widely conceptualised as a deliberate and structured process through which individuals critically examine experience to generate learning and inform future action. Dewey (2011) described the reflection process as an individual reviewing their experiences to evaluate them and subsequently changing the practice or understanding. In professional practice, reflection moves beyond simple recollection, involving analysis of thoughts, emotions, assumptions and contextual factors that shape behaviour. Early work by Schön (1983) distinguished between *reflection-in-action*, which occurs during practice, and *reflection-on-action*, which takes place retrospectively, emphasising reflection as a core professional skill in complex and uncertain environments such as healthcare.

Building on this, Gibbs (1988) proposed a cyclical model of reflection that structures reflective thinking through sequential stages: description, feelings, evaluation, analysis, conclusion and action planning. This model has been widely adopted in nursing education because it scaffolds reflective thinking and makes the process accessible, particularly for practitioners developing reflective capability. Similarly, Boud et al. (1985) emphasised the role of emotions and experience in learning, arguing that reflection is most effective when individuals attend not only to cognitive evaluation but also to affective responses, recognising feelings as integral to meaning-making and professional growth.

Johns (2017) further advanced reflective practice within nursing by situating reflection explicitly within ethical, personal and empirical dimensions of practice where the model encourages practitioners to interrogate how internal values, external influences and patterns of knowing interact within lived experience. Across these models, reflection is consistently understood as a purposeful, developmental activity that supports learning, identity formation and adaptive practice, particularly in periods of transition, uncertainty or change.

These reflective models are widely embedded in international nursing education and curricula as a means of developing reflective capability (Barchard, 2022). Reflective practice is a core component of preregistration nursing proficiencies and professional standards across multiple jurisdictions, including the United Kingdom (NMC, 2018a, 2018b), Australia (ANMAC, 2025), New Zealand (NCNZ, 2024) and the United States (AACN, 2021), and is integral to ongoing professional development and revalidation (NMC, 2019a). In Nordic countries such as Denmark, reflection is formally recognised as a professional competence within ministerial regulations and nursing curricula, with ethical guidelines explicitly endorsing reflective practice as fundamental to the profession (BEK nr 978 af 23/06/2022).

Reflection supports nurses, midwives and nursing associates to critically examine practice, identify learning and implement improvements, thereby enhancing professional accountability, teamworking and patient outcomes (Nursing and Midwifery Council: NMC, 2019, 2021). In countries with Indigenous populations, reflective practice is closely aligned with cultural safety. In New Zealand, Kawa whakaruruhau is a core requirement within nursing standards and competencies (Nursing Council of New Zealand: NCNZ, 2024), whereas in Australia culturally safe practice particularly in relation to Aboriginal and Torres Strait Islander peoples is mandated in law. However, limitations have been identified in the availability of effective reflective practice supports to enable culturally safe outcomes (Nursing and Midiwifery Board of Australia: NMBA, 2018; Wilson et al., 2024). In the United States, self-reflection is explicitly positioned within Domain 10 (of the American Association of Colleges of Nursing [AACN] curriculum essential document) as central to personal, professional and leadership development (AACN, 2021). This paper is however limited by the absence of perspectives from African, Asian and Eastern European contexts, highlighting the need for greater inclusion of voices from the global majority in future work.

## Reflective practice in menopause transition

Menopause is increasingly recognised as a biopsychosocial transition involving embodied change, identity renegotiation and shifts in relational and professional roles (Blais, 2024). Qualitative research consistently demonstrates that women experience menopause not simply as a biological event, but as a period of time requiring reinterpretation of bodily signals, professional competence and social expectations (Cronin et al., 2021, 2023; GOV.UK, 2025). Reflective practice offers a structured means of supporting this meaning-making process, enabling individuals to contextualise fluctuating symptoms, recognise patterns over time and reduce self-blame, particularly in cultures where menopause remains under-recognised or stigmatised.

Within nursing, reflective practice is well established as a mechanism for clinical learning, ethical reasoning, professional development and emotional processing (Bulman and Schutz, 2013; NMC, 2021). Reflection supports attention to the cognitive, emotional and contextual dimensions of experience and is particularly valuable in situations characterised by complexity, uncertainty and change. Evidence from nursing and healthcare contexts suggests that reflective practice enhances self-awareness, supports clinical decision-making and contributes to practitioner well-being. Structured individual and group reflective forums have been shown to reduce isolation, emotional burden and moral distress, with relevance for nurses managing menopause alongside demanding professional roles (Horton-Deutsch and Sherwood, 2023; Ng et al., 2023; Wilson et al., 2025a).

Evidence from nursing and occupational health literature further indicates that reflective practices can reduce menopause-related psychological distress in workplace settings. Individual and group-based reflective dialogue supports emotional processing, validation of experience and adaptive coping, while reducing isolation and enhancing self-efficacy (Bazeley et al., 2022; Hardy et al., 2018). This is especially important given that many women minimise or normalise menopausal symptoms to preserve professional identity and performance (Atkinson et al., 2021).

From an evidence-based perspective, reflective practice aligns with non-pharmacological approaches recommended for menopause management, including cognitive behavioural strategies, mindfulness and psychosocial support. Menopause-specific cognitive behavioural therapy explicitly incorporates reflective components such as symptom monitoring, cognitive appraisal and values-based action planning (Denton et al., 2025). Reflective practice therefore functions both as a therapeutic mechanism and as a transferable skill, supporting autonomy, self-management and shared decision-making (Horton-Deutsch and Sherwood, 2023).

Applied to menopause, reflection enables women to identify patterns or triggers in symptoms, distinguish between transient stress-related effects and concerns requiring further investigation or referral (e.g. thyroid dysfunction) and engage in values-based decision-making regarding treatment options such as hormone replacement therapy, psychological interventions, lifestyle modification or watchful waiting, consistent with United Kingdom National Institute of Clinical Excellence (NICE) guidance on individualised care (NICE, 2015; updated 2024). Reflective approaches also support proactive planning for workplace adjustments, caring responsibilities and career decisions, reducing reliance on reactive crisis management (Wilson et al., 2025a). Crucially, reflective practice provides a protected space to process emotions commonly associated with menopause including loss, altered body image, grief, shame and uncertainty that are frequently overlooked in routine clinical encounters (Blais, 2024).

## Impact of menopause in the workplace

An increasing number of women are working through menopause, and women constitute a substantial proportion of the health and social care workforce (Bazeley et al., 2022; Brewis et al., 2017; Royal College of Nursing: RCN, 2023). For many, the menopause transition coincides with mid- to late-career stages, when they are experienced practitioners holding senior, leadership or management roles. Others encounter menopause alongside career change or entry into new professional roles, compounding adjustment demands. Together with responsibilities outside of work such as relationships, personal health concerns and caregiving for children or ageing relatives, these intersecting demands create cumulative pressures that can amplify the physical, emotional and professional challenges of menopause.

Healthcare workplaces are characterised by high workload, rapid decision-making and complex patient needs. Within this context, international evidence highlights significant workforce retention challenges, with experienced nurses and healthcare professionals disproportionately reducing hours or exiting employment (Cronin et al., 2023, 2024; GOV.UK, 2025). Survey data indicate that menopausal symptoms contribute materially to these trends: one in ten women report leaving employment due to menopausal symptoms, whereas over two-thirds report a negative impact on work performance. A substantial minority consider leaving their roles due to inadequate workplace support, and experiences of discrimination remain common (CIPD, 2023; Fawcett Society, 2025).

These findings suggest that menopause is not only a personal health issue but also a workplace and workforce sustainability concern. Emerging evidence indicates that reflective practice may function as a supportive buffer by enabling sense-making, validation and adaptive coping in work contexts. Digital and structured reflective tools, in particular, offer a scalable option that can be accessed flexibly by nurses and employers, aligning with contemporary workforce support strategies (Bembridge et al., 2025).

## Aligning reflective practice with menopause support needs

The menopause transition frequently intersects with peak professional responsibility, caregiving demands and organisational expectations. When symptoms remain unacknowledged or unsupported, they are associated with absenteeism, presenteeism, reduced confidence and increased attrition, particularly within female-dominated professions such as nursing (Cronin et al., 2023, 2024). Organisational reviews and parliamentary inquiries in the United Kingdom and Australia have documented the personal, economic and equality impacts of inadequate menopause support, highlighting the costs of silence, stigma and delayed workplace adjustment (Chartered Institute of Personnel and Development: CIPD, 2023; House of Commons Women and Equalities Committee, 2022; Department of Health & Social Care, 2022; GOV.UK., 2021, 2025).

Evidence also demonstrates that menopause support is unevenly distributed across populations. In Aotearoa New Zealand, Māori women experience barriers to culturally appropriate menopause care, including lower access to hormone replacement therapy, higher prescribing of antidepressants, and limited recognition of rongoā Māori within Te Ao Māori frameworks of health and well-being (Bullivant et al., 2021; Pihama, 2020). These inequities underscore the need for menopause support approaches that are culturally responsive, relational and grounded in reflective engagement rather than purely biomedical models.

Reflective practice is therefore well positioned as both a clinical and organisational support strategy within menopause care. It aligns with policy emphases on personalised care, shared decision-making, partnership working and psychosocial and spiritual well-being, while offering a low-cost, adaptable approach that can be embedded within existing professional development, supervision or group learning structures. By legitimising experience, facilitating dialogue and supporting adaptive action, reflective practice has the potential to enhance individual well-being while contributing to workforce retention, equality and compassionate leadership across health and social care systems.

At the same time, the preceding analysis highlights the limitations of existing approaches. Although reflective practice is widely endorsed in nursing, available models are not designed to address the specific complexities of the menopause transition, nor do they consistently integrate embodied experience, workplace context and health system navigation. In addition, this analysis is limited by the absence of African, Asian and Eastern European perspectives, reflecting broader gaps in the menopause and workforce literature and highlighting the need for adaptable, culturally responsive frameworks informed by diverse experiences.

## An integrated framework for menopausal reflection: the MIRROR Cycle

A wide range of reflective practice models are well established in nursing and allied professions. However, no single model adequately addresses the complexity of the menopause transition, which encompasses embodied symptoms, emotional and identity shifts, workplace dynamics and healthcare navigation. Drawing on an established programme of work on menopause and the nursing workforce including literature reviews, empirical research, policy and education, we developed an integrated, person-centred reflective framework, tailored specifically to menopausal experience.

The MIRROR Cycle synthesises foundational reflective models (Boud et al., 1985; Gibbs, 1988; Johns, 2017; Schön, 1983) and adapts them to the specific aims of menopausal reflection: meaning-making, symptom mapping, action planning and advocacy. The framework has been informed by interdisciplinary and international collaboration among nurse educators and researchers, alongside engagement with nurses’ lived experiences of menopause in clinical, educational and workplace contexts.

The framework is underpinned by five core principles that support its application across nursing education, clinical practice and organisational settings:

1. Non-judgemental stance: Reflection begins from a compassionate, curiosity-driven approach to reduce shame and self-blame. When facilitated by others, it requires openness, empathy and psychological safety.
2. Situational and longitudinal lenses: The framework integrates immediate, event-focused reflection (e.g. a hot flush during a meeting) with periodic life-course reviews (e.g. monthly or quarterly), enabling pattern recognition that supports ‘talk, track and treat’ approaches (Cronin et al., 2023).
3. Multidimensional data modalities: Reflection incorporates embodied (symptom diaries), cognitive (thought records), social (relationships and workplace interactions) and clinical data (medications and investigations), alongside non-clinical and holistic practices.
4. Action orientation: Reflection is directed towards practical outcomes, supporting micro-adjustments in daily practice and escalation planning where symptoms or concerns persist.
5. Social embedding: The framework promotes peer reflection and organisational structures that normalise disclosure, enable dialogue and support reasonable workplace adjustments.

**Figure 1.**
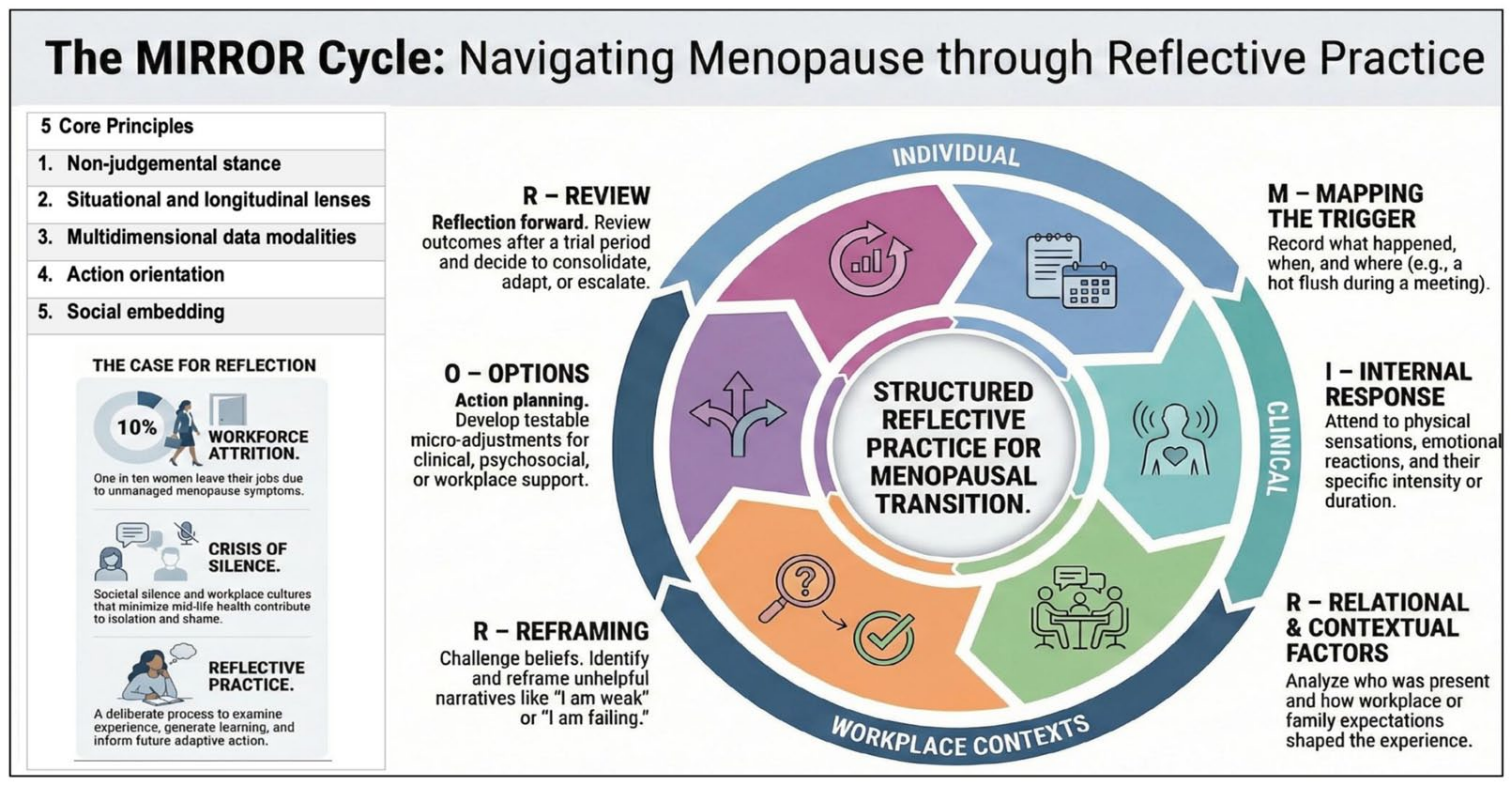
MIRROR Cycle, an adapted reflective cycle for navigating menopause (Cronin et al., 2026).

Building on these principles, the MIRROR Cycle was developed, a six-step reflective process that integrates descriptive, emotional, analytical and action-oriented reflection, with explicit attention to workplace context and service navigation. The developmental logic of the MIRROR Cycle and its alignment with the five core principles are outlined in Table 1 and illustrated in Figure 1.

**Table 1.** Mapping the steps of menopause reflection in the MIRROR Cycle (Cronin et al., 2026).

MIRROR step	Mapped from menopause reflective cycle	Description
M – Mapping the trigger	Trigger and describe	What happened? When and where did it occur? Initial description of the menopausal experience or event (e.g. sleep disruption, hot flush at work, emotional response in a meeting).
I – Internal (embodied) response	Embodied response	Physical sensations, emotional reactions, intensity and duration. Attends explicitly to the body and affect.
R – Relational and contextual factors	Contextual mapping	Exploration of explanatory narratives, assumptions, identity concerns, stigma, self-judgement; identification of unhelpful cognitions.
R – Reframing meaning and beliefs	Meaning and beliefs	Exploration of explanatory narratives, assumptions, identity concerns, stigma, self-judgement; identification of unhelpful cognitions.
O – Options and action planning	Options and planning	Identification of practical, clinical, psychosocial and workplace options; development of a small, testable action plan.
R – Review and reflection forward	Review and share	Reviewing outcomes, consolidating learning, adapting or escalating actions, and sharing safely.

This cyclic approach maintains alignment with nursing reflective traditions that emphasise learning, professional growth and improved practice, while extending reflection to address menopausal experience explicitly. The MIRROR Cycle can be applied at individual, team and organisational levels. As shown in Figure 1, the cycle flows clockwise through six stages: mapping the trigger; identifying internal (embodied) responses; exploring relational and contextual factors; reframing meaning and beliefs; generating options and action plans and reviewing learning and next steps.

The MIRROR Cycle has been refined for use across the menopause journey and is adaptable for individual self-reflection, clinical consultations and group reflective forums such as Schwartz Rounds and reflective supervision. These approaches provide structured opportunities to pause, take stock and reflect safely. Effective reflection requires time, facilitation and prompts within psychologically safe environments. Box 1 therefore outlines practical strategies for integrating menopause-focused reflective practice into education, clinical settings and workplace support structures.

**BOX 1.** Strategies at individual, team and organisational level.

**1. Individual strategies** *a. Symptom and reflection journal* Keep a simple daily/weekly log combining symptom tracking (timing, severity, triggers), sleep and mood, plus a short reflective entry using the MIRROR Cycle prompts. Evidence supports reflective journaling for increasing self-awareness and reducing burnout in healthcare workers. Practical format: 3–5 lines per day, plus a weekly 15–30-minute review. *b. Brief structured reflection (‘10-minute MIRROR Cycle slot’)* Set a scheduled micro-reflection (10 minutes) once or twice weekly: describe a trigger, note feelings, write one coping option and one action (e.g. book general practitioner (GP) appointment; request a temp-control fan at work). The brevity increases likelihood of sustained use. *c. Narrative work and reframing* Use narrative prompts (‘What story am I telling myself about this change?’) to identify limiting beliefs. Cognitive reframing and acceptance approaches are often used with CBT interventions recommended by NICE as part of supportive care. *d. Mindfulness and body-scanning* Short, guided body scans can reconnect a woman to her embodied experience and reduce automatic shame responses; these are complementary to reflective writing, not replacements.
**2. Peer and professional support strategies** *a. Reflective supervision/coaching* One-to-one reflective supervision (mentor or occupational health) offers a confidential space to process emotions and plan actions. Reflective supervision has a strong tradition in nursing and allied professions (Horton-Deutsch and Sherwood 2023). *b. Schwartz Rounds and facilitated group reflection* Schwartz Rounds (Maben et al., 2021; Ng et al., 2023) are structured sessions where staff reflect on the emotional and social sides of caregiving; evidence shows they reduce isolation, increase insight and support well-being. They can be adapted to include menopause narratives and cross-discipline participation. Implementing rounds or menopause-focused reflection groups can normalise disclosure and reduce stigma. *c. Peer and/or whanau (culturally connected family/community) support groups and guided reflective circles* Peer groups (online or workplace) offer normalisation and facilitated circles using the MIRROR Cycle prompts can safely surface workplace drivers of distress and co-produce reasonable adjustments (Sherwood et al., 2025). Peer and or whanau support groups are very important in Indigenous cultures where being part of a collective culture is integral to identity with (for example) Māori (New Zealand) women able to form and be part of mana (empowered) wahine (women) support groups and Aboriginal and Torres Strait Islander women (Australia) gaining valuable support through yarning circles (Wilson et al., 2024). *d. Reflective practice tools integrated into health encounters* Clinicians can use the MIRROR Cycle as a consultation template: brief symptom mapping, reflective exploration of impact and shared action planning (Hormone Replacement Therapy: HRT, sleep hygiene, Cognitive Behavioural Therapy: CBT referral). This aligns with NICE (2024) recommendations for individualized discussion and shared decision-making.
***3. Organisational strategies*** *a. Manager training in reflective leadership and menopause literacy* Managers trained to hold brief reflective conversations (listening, noting impact, agreeing adjustments) create psychologically safe environments. Equality and Human Rights Commission: EHRC (2024) guidance emphasises manager capability as central to supportive workplaces. *b. Policy and practical adjustments* Clear menopause policies, temperature control, flexible working, rest spaces and access to occupational health are concrete outputs once reflective discussions identify needs. In the United Kingdom, the Department of Health and Social Care (2022) Women’s health strategy is supported by a range of organisations to provide guidance and practical checklists for organisations. Similarly in New Zealand, workplace legislation ensures women experiencing menopause must be supported at work with several organisations providing useful guides for offering support for example Vodafone and BUPA. *c. Reflective learning embedded in staff well-being programmes* Integrate monthly reflective sessions for example Schwartz Rounds (Maben et al., 2021; Ng et al., 2023) and action learning sets ensuring confidentiality, skilled facilitation and pathways for practical follow-up occupational health referrals and adjustments (EHRC, 2024).

## Discussion

### Why an applied reflective framework is needed

Menopause is widely recognised as a complex biopsychosocial transition shaped by symptom burden, social stigma, service variability and gendered ageism across healthcare and workplace contexts. These intersecting pressures are compounded by midlife role demands, caregiving responsibilities and cumulative health risks, contributing to disruption in daily functioning, professional identity and workforce participation. Although this context is well-documented, less attention has been paid to *how* clinicians, educators and organisations can support women to navigate menopause in practical, structured and equitable ways. The MIRROR Cycle responds to this gap by translating reflective practice from a conceptual ideal into an applied, action-oriented tool.

### Implementing the MIRROR Cycle in practice

The MIRROR Cycle is designed for flexible use across individual, clinical, educational and organisational settings. At the individual level, it supports women to map symptoms, recognise patterns and translate insight into manageable actions. In clinical consultations, it offers a structure for shared exploration of embodied experience, treatment options and service navigation within time-limited appointments. In group contexts such as reflective supervision, Schwartz Rounds, or peer forums, it provides a psychologically safe scaffold for collective sense-making, validation and advocacy.

Importantly, the MIRROR Cycle aligns with existing reflective traditions in nursing while extending them to explicitly address menopause, workplace context and health system interaction. Its cyclical design encourages both immediate reflection on specific triggers and longitudinal review across the menopause transition, supporting continuity, self-efficacy and proactive planning rather than crisis-driven responses.

### The MIRROR Cycle as a strategic clinical tool: an illustrative scenario

To illustrate its application, consider a senior nurse experiencing disrupted sleep, cognitive fog and anxiety during shift handovers. Using the MIRROR Cycle, she first maps the trigger (handover meetings following night shifts), then explores internal responses (fatigue, reduced concentration and self-doubt). Reflection extends to relational and contextual factors, including workload expectations, staffing shortages and fear of being perceived as less competent. Through guided reframing, she identifies internalised stigma and unrealistic self-expectations shaped by gendered workplace norms.

The options and action planning phase supports practical steps: requesting temporary shift adjustments, accessing menopause-informed clinical advice and engaging in peer reflective supervision. Review of outcomes enables iterative adjustment and reinforces learning. In this way, reflection functions not only as emotional processing but also as a strategic clinical and organisational tool that supports well-being, performance and retention.

### Preparing clinicians and educators to use menopause-focused reflection

Effective use of the MIRROR Cycle requires preparation and confidence among clinicians, educators and facilitators. Historically, menopause education within undergraduate and postgraduate nursing and primary care curricula has been inconsistent, contributing to variable confidence and practice (Anto et al., 2025). Integrating menopause-specific reflective frameworks into education, supervision and continuing professional development can strengthen clinicians’ capacity to support shared decision-making, values-based care and personalised treatment discussions (Panay et al., 2025), including CBT and other non-pharmacological approaches recommended in updated NICE guidance (NICE NG23, 2024).

### Advocating for organisational and policy change

At an organisational level, the MIRROR Cycle offers a mechanism to operationalise menopause policy commitments. Data that are aggregated ethically and anonymously can inform workforce planning, reasonable adjustments and service redesign (Atkinson et al., 2021). This is particularly relevant given growing legal accountability around menopause-related discrimination and the persistent gap between policy intent and lived experience. Embedding reflective practice within occupational health, leadership development and equality strategies can support cultures of openness, early intervention and compassionate leadership.

### Equity, cultural safety and the limits of reflection

Although reflective practice can enhance agency and self-advocacy, it cannot compensate for structural inequities. Cultural invisibility and access to menopause care and workplace support varies significantly by ethnicity, socio-economic position and geography (Ajayi et al., 2025). Indigenous and ethnically diverse women remain under-served, reflecting broader epistemological and systemic neglect (Gore & Morgan, 2025). The MIRROR Cycle must therefore be implemented within culturally safe frameworks and alongside structural reforms; reflection alone is insufficient where services, funding or culturally appropriate care are absent.

### Implications for practice, education and research

This paper positions the MIRROR Cycle as a practical, transferable reflective framework that bridges personal experience, clinical care and organisational responsibility. Future research should evaluate its implementation across diverse settings, explore digital and face-to-face adaptations and centre the voices of women from the global majority (Cronin et al., 2025). For nursing practice and leadership, the MIRROR Cycle offers a means to transform reflection into a strategic tool for improving menopause care, workforce retention and gender-equitable workplaces.

### Recommendations

These recommendations are directed at clinicians, nurse educators, employers and policymakers. They focus on embedding structured reflective practice, specifically the MIRROR Cycle as a strategic tool within menopause care, workplace support and professional education.

1. Clinical practice: embedding reflective structure into menopause care

In clinical settings, menopause support should integrate reflective approaches longitudinally rather than as isolated conversations. Using a structured tool such as the MIRROR Cycle enables consultations to function as both therapeutic encounters and self-management interventions, supporting symptom mapping, meaning making and action planning over time. This approach aligns with ‘talk, treat and track’ models of care (Cronin et al., 2023) and facilitates shared decision-making within time-limited appointments.

Brief reflective tools such as symptom diaries, structured prompts or digital applications can support ongoing monitoring and review, whereas reflective supervision for clinicians delivering menopause care is essential to process emotional labour, mitigate burnout and enhance clinical judgement. Evidence indicates that structured reflective forums, including Schwartz Rounds, reduce isolation and support professional well-being (Maben et al., 2021; Ng et al., 2023). Continuing professional development should therefore integrate menopause literacy, reflective skills and cultural competence, co-produced with women with lived experience to ensure relevance and applicability.

2. Workplace implementation: reflection as an organisational support mechanism

Employers play a critical role in translating reflective insight into practical workplace adjustments. Managers should receive training in menopause guidelines and toolkits that include brief reflective conversations, enabling needs to be identified, normalised and followed by action. Clear routes for confidential disclosure and escalation should be embedded within organisational processes, supported by guidance from bodies such as CIPD (2023) and EHRC (2024).

Organisations should establish regular, psychologically safe reflective forums such as menopause circles, Schwartz-style sessions or facilitated peer reflection linked directly to occupational health services and reasonable adjustments. Digital reflective tools can complement face-to-face options, supporting accessibility and self-management (Cronin et al., 2023), whereas practical adjustments (e.g. flexible working, temperature control and rest breaks) should routinely follow reflective needs assessments rather than requiring crisis-driven advocacy by individuals.

3. Health systems and policy: enabling reflective, equitable care

Health services and policymakers should standardise menopause education and care pathways in line with updated NICE guidance (NICE NG23: 2024), ensuring that reflective, shared decision-making is recognised, resourced and evaluated as part of routine care. Multidisciplinary menopause services should be co-designed with women, particularly those with complex needs and equity of access monitored across ethnicity, socio-economic position and geography (Conquer et al., 2026).

Workforce and health outcome data such as retention, sickness absence and service utilisation should be collected in relation to menopause support initiatives, strengthening the economic and policy case for sustained investment. Parliamentary and regulatory reports increasingly identify menopause as a workforce and equality issue; reflective practice provides a practical mechanism to operationalise these policy commitments (Cronin et al., 2024; EHRC, 2024).

4. Research and implementation priorities

Future research should rigorously evaluate menopause-specific reflective interventions, including the MIRROR Cycle, comparing approaches such as guided journaling, reflective supervision and group forums against usual care. Outcomes should include symptom burden, help-seeking behaviour, workplace retention, well-being and equity of access.

Research methodologies must extend beyond Western paradigms and incorporate culturally safe and gender-informed frameworks, particularly for Indigenous and ethnically diverse populations. Importantly, reflection should be evaluated not only as an individual coping strategy but also as a system-enabled intervention whose effectiveness depends on organisational and policy contexts.

5. Safeguards for implementation

Successful implementation requires careful attention to confidentiality, psychological safety and facilitation quality. Reflective forums should be moderated by trained facilitators with clear ground rules, escalation pathways and safety-netting for participants experiencing significant distress (Thavabalan et al., 2025). Managerial capacity must be supported through protected time, training and incentives. Reflective approaches should complement not replace medical assessment and evidence-based treatment, including hormone replacement therapy when appropriate, alongside psychological and workplace supports consistent with NICE guidance (NICE NG23: 2024).

## Conclusion

The menopause transition is a complex, identity-laden life stage that spans clinical, social and occupational domains and is frequently experienced as isolating. When reflective practice is adapted into structured, action-oriented frameworks such as the MIRROR Cycle, it can function as a strategic clinical and organisational tool supporting self-management, informing decision-making and enabling timely workplace and healthcare responses.

Reflective practice is not a substitute for evidence-based clinical care or structural change in health services and workplaces. However, when embedded alongside these measures, it offers a low-cost, high-value approach to restoring agency, reducing isolation and translating lived experience into meaningful action. Investment in reflective strategies at individual, team and organisational levels has the potential to improve health outcomes, workforce retention and equity.

Nursing, positioned at the intersection of clinical care, education and workplace well-being, is uniquely placed to lead menopause-informed reflective practice in partnership with women with lived experience. Future research should evaluate the effectiveness and equity of reflective interventions across diverse settings. By embedding structured reflection into everyday practice, nurses and nurse leaders can help transform reflective time into tangible, person-centred change during the menopause transition.

**Key points for policy, practice and research**

- Menopause significantly affects nurses’ well-being, work performance and retention, yet remains under-recognised in practice, education and policy.
- Structured reflective practice provides a practical, low-cost and scalable approach to menopause support, helping nurses make sense of symptoms, engage in self-management and navigate workplace and clinical challenges.
- The MIRROR Cycle operationalises menopause-informed reflection, enabling nurses to map triggers, explore embodied and relational responses, plan actionable steps and review outcomes at individual, team and organisational levels.
- Embedding menopause-aware reflection within clinical practice, education, supervision and organisational policy promotes inclusive, culturally safe and gender-responsive care while reducing stigma and supporting workforce sustainability.
